# Exploring the relationships between hormone receptor, HER2 status, and bone involvement in the first distant metastases of in Chinese breast cancer patients who lacked HER2 targeted therapy

**DOI:** 10.1186/s12885-023-10569-z

**Published:** 2023-01-26

**Authors:** Zhifeng Jia, Muwei Dai, Yuguang Shang, Yue Li, Zhensheng Li

**Affiliations:** 1 Department of Orthopedic Surgery, The Fourth Hospital of Hebei Medical University, Shijiazhuang, 050035 China; 2 Department of Radiation Oncology, The Fourth Hospital of Hebei Medical University, 169 Tianshan Street, Shijiazhuan, 050035 China; 3 Department of Medical and Radiation Oncology, Hebei Yiling Hospital, Shijiazhuang, 050011 China

**Keywords:** Breast cancer, Bone metastasis, Hormone receptor, HER2, Endocrine therapy

## Abstract

**Background:**

This retrospective study explored the relationship between hormone receptor (HR), human epidermal growth factor receptor 2 (HER2) status, and bone involvement in the first distant metastases (DM) of Chinese breast cancer (BC) patients who lacked the HER2 targeted therapy. Such therapy was rarely received due to its lag approval or high cost in China compared with the developed countries.

**Methods:**

All eligible women with primary unilateral stage I – III BC and first DM diagnosed in 2008—2018 at one cancer center were identified for enrollment. Based on chart records, a full or no/partial compliance status of endocrine therapy (ET) was assigned for HR-positive patients. Multivariate logistic regression was used to estimate the adjusted odd ratio (aOR), its 95%CI and *p* value.

**Results:**

Four hundred eighteen patients had an average age of 50.7 years and median disease-free survival of 27.1 months at DM. Bone, lung, liver and brain metastasis rates in patients were 55.7%, 34.7%, 33.0% and 8.1%, respectively. Compared to HR-negative patients, HR-positive patients with the full and non/partial compliance of ET were significantly associated with higher risk of bone involvement with an aOR of 2.329 (1.316 – 1.741, *p* = *0.004*) and 2.317 (1.330 – 4.036, *p* = *0.003*), respectively. No difference of such risk was found between the two groups of ET compliance (*p* = *0.984*) nor between HER2-negative and HER2-positive patients (aOR 0.827, *p* = *0.431*). Stratified analyses further indicated that HR-positive was associated with bone involvement only in HER2-negative BC patients (*p* = *0.006—0.015*).

**Conclusions:**

HR-positive tumors are significantly associated with bone involvement in HER2-negative metastatic BC patients. ET does not appear to impact this association. HER2 status per se is not associated with such risk.

## Background

Bone is the most common site of distant metastasis (DM) in breast cancer (BC) [1, 2]. Once bone is metastasized, BC becomes incurable. However, patients with bone-only metastasis still have a median survival of 2 - 3 years [3]. Many clinical trials have established the effectiveness of endocrine therapy (ET), human epidermal growth receptor 2 (HER2) targeted therapy, and bone modifying agents (bisphosphonates and denosumab) in preventing BC progression [4–7]. So, it becomes intriguing to determine the clinical relationships of estrogen receptor (ER), progesterone receptor (PR), and HER2 with first bone metastasis (BM). The preferential BM of BC is a complex of numerous interactive factors when the circulatory tumor cells are deposited in bone [8, 9]. Through those factors, the ER-positive (ER+) tumor has consistently been reported to be associated with higher BM risk while the lack of hormone receptor (HR, ER or PR) is associated with visceral recurrence [10–12]. However, the therapeutic roles of ET and HER2 targeted drugs in HR-positive (HR+) or HER2-positive (HER2+) patients in establishing these links were mostly unknown.

In China, Trastuzumab was the first HER2 targeted drug and was approved for use in 2002 and its administration rate in HER2+ patients was reported to be only 20% in the Beijing area in 2008. Slowly, it became a common practice but still many years after the developed countries [13, 14]. As a result, the majority of metastatic Chinese BC patients during a period of time did not benefit from any HER2 targeted therapy before DM [13, 14]. Meanwhile, a large proportion of HR+ patients had not faithfully taken the prescribed ET drugs for many reasons [13]. Considering those factors, we designed an epidemiological study of BC patients with first DM in order to explore the relationships of bone involvement of DM with the HR and HER2 status. Perhaps, this study’s results could assist the hypothesis generation of clinical research on BM, and help guide better use of bone imaging and bone modifying agents in BC patients.

## Methods

### Patients and study design

All 425 women with primary unilateral stage I – III BC and mastectomy with first DM in 2008 – 2018 at our institution were identified. Other patient’s inclusion criteria included: (1) BC diagnosis age ≥ 18 years and calendar year 1997 or later, and (2) mastectomy and axillary lymph node dissection on the primary tumor being treated. Very few patients (*n* = 3) had breast conservative surgery and/or sentinel lymph node biopsy. They were purposely excluded to simplify the adjustment analysis, avoid the possible high standard error of statistical regression coefficient, and make interpretation of the results straightforward. Seven patients (1.6%) had Trastuzumab treatment and were excluded from analysis as well. Patient’s other exclusion criteria included: (1) primary inflammatory or bilateral BC (2) DM diagnosed before the completion of surgery, chemotherapy, or radiotherapy (RT) for the primary tumor, and (3) metastasis tumor likely from other malignant disease.

When designing this study, we carefully chose the study population and analysis strategies. We intentionally excluded the patients with locoregional recurrence (LRR) who were defined to have an ipsilateral or contralateral recurrence on their chest wall or regional lymph nodes (i.e. axillary, supraclavicular, subclavian, internal mammary ones). We considered the LRR to have a different main mechanism from DM. Since we believed that the timing of BM detection was sensitive to the follow-up schedule, patient symptoms, and imaging tools, we decided to use the logistic regression to explore the relationship.

This study was approved by the Research Ethics Committee of the Fourth Hospital of Hebei Medical University (FHHMU # 2020-188). All study patients were provided with the written informed consent. All sensitive health information of patients was excluded from the study dataset.

### Determination of biomarker and ET compliance

The biomarkers of primary tumor were utilized. HR+ was defined as ER+ or PR+ (both criteria were ≥ 1% by the IHC method). HER2+ was defined as the IHC 3+ result or the fluorescence situ hybridization-positive (FISH+) on IHC 2+ tumor. HER2-negative (HER2−) was defined as either IHC 0/1+ or FISH-negative (FISH−) on IHC 2+ tumor. HER2-undetemined was referred to as the IHC2+ result without the FISH test.

Two ET compliance statuses were summarized from medical records – the full or non/partial compliance – for the HR+ patients. Per charts, only oral ET drugs (Tamoxifen, Toremifene, Letrozole, Anastrozole and Exemestane) were noticed to be used. The full compliance of ET was defined as the HR+ patients who took the prescribed drugs faithfully until DM diagnosis or at least 5 years prior to DM. No ovarian removal procedure or ovarian function suppression (OFS) drugs or bone modifying agents were taken before DM.

In analysis, the endpoint of binary bone involvement status of DM was analyzed. The categorical disease-free survival (DFS) and other conventional baseline variables including BC age, tumor characteristics and treatments were considered as covariate candidates. The final covariates in multivariate model were determined from the univariate analysis result and literature review.

### Statistical methods

Continuous and categorical variables were summarized by the descriptive statistics. For the comparison or relationship examination between subgroups, analysis of variance (ANOVA), Chi-square or Fisher’s exact tests, when appropriate, were used. Univariate and multivariate linear logistic regression models were used to evaluate the odd ratio (OR), its 95% confidence interval (CI) and associated *p* value. Two-sided *p* < *0.05* was cited as the statistical significance level. All analyses were performed with SAS 9.4 for Windows.

## Results

### Patient baseline characteristics

The analyzed 418 patients had an average age of 50.7 years and a median DFS of 27.1 months at DM. Their bone, lung, liver and brain metastasis rates were 55.7%, 34.7%, 33.0% and 8.1%, respectively. Bone-only, bone and others, others-only metastasis patients were 14.6%, 41.1%, and 44.3%, respectively. Table 1 shows the baseline characteristics of these patients. In brief, 53.3% of patients had a primary tumor located in their left breast and 77.5% of patients had their tumor in an external quadrant. 70.8% of patients had a T2 or larger tumor. Patients with metastatic locoregional lymph nodes (N1-3) had a high prevalence of 64.1%. 70.8% of patients were HR+ patients and 35.6% were HER2+. Lastly, 95.7% of patients had chemotherapy and 44.7% had radiotherapy (RT).

**Table 1:** Baseline characteristics of patients

Variable	All (n, %)	Bone involvement at DM (n, %)		*P*^a^
		No	Yes	
Patients	418(100.0)	185( 45.7)	233( 55.7)	
BC diagnosis age (years) mean ± std	47.9 ± 10.0	48.6 ± 10.4	47.3 ± 9.6	*0.181*
< 40	88( 21.1)	35( 18.9)	53( 22.7)	*0.536*
< 50	144( 34.4)	61( 33.0)	83( 35.6)	
< 60	135( 32.3)	63( 34.1)	72( 30.9)	
≥ 60	51( 12.2)	26( 14.1)	25( 10.7)	
BC diagnosis year ^b^				
1997 ~ 2010	169( 40.4)	74( 40.0)	95( 40.8)	*0.873*
2011 ~ 2016	249( 59.6)	111( 60.0)	138( 59.2)	
Tumor laterality				
left	223( 53.3)	98( 53.0)	125( 53.6)	*0.891*
right	195( 46.7)	87( 47.0)	108( 46.4)	
Tumor quadrant				
internal	94( 22.5)	37( 20.0)	57( 24.5)	*0.278*
external	324( 77.5)	148( 80.0)	176( 75.5)	
T stage				
T1	97( 23.2)	42( 22.7)	55( 23.6)	*0.374*
T2	220( 52.6)	102( 55.1)	118( 50.6)	
T3-4	76( 18.2)	34( 18.4)	42( 18.0)	
unknown	25( 6.0)	7( 3.8)	18( 7.7)	
N stage				
N0	138( 33.0)	57( 30.8)	81( 34.8)	*0.233*
N1-3	268( 64.1)	125( 67.6)	143( 61.4)	
unknown	12( 2.9)	3( 1.6)	9( 3.9)	
Tumor pathology				
IDC	345( 82.5)	151( 81.6)	194( 83.3)	*0.661*
other	73( 17.5)	34( 18.4)	39( 16.7)	
Tumor grade				
I-II	267( 63.9)	118( 63.8)	149( 63.9)	*0.716*
III	89( 21.3)	37( 20.0)	52( 22.3)	
unknown	62( 14.8)	30( 16.2)	32( 13.7)	
LVI				
positive	94( 22.5)	42( 22.7)	52( 22.3)	*0.992*
negative	105( 25.1)	46( 24.9)	59( 25.3)	
unknown	219( 52.4)	97( 52.4)	122(52.4)	
HR				
positive	296( 70.8)	116( 62.7)	180( 77.3)	*0.001*
negative	117( 28.0)	68( 36.8)	49( 21.0)	
unknown	5( 1.2)	1( 0.5)	4( 1.7)	
HER2				
positive	149( 35.6)	70( 37.8)	79( 33.9)	*0.220*
negative	219( 52.4)	90( 48.6)	129( 55.4)	
undetermined	35( 8.4)	20( 10.8)	15( 6.4)	
missing	15( 3.6)	5( 2.7)	10( 4.3)	
Chemotherapy				
yes	400( 95.7)	176( 95.1)	224( 96.1)	*0.616*
no	18( 4.3)	9( 4.9)	9( 3.9)	
RT				
no	231( 55.3)	105( 56.8)	126( 54.1)	*0.584*
yes	187( 44.7)	80( 43.2)	107( 45.9)	
HR and ET compliance status				
HR-positive and full one	163( 39.0)	63( 34.1)	100( 42.9)	*0.004*
HR-positive and no/partial one	133( 31.8)	53( 28.6)	80( 34.3)	
HR-negative	117( 28.0)	68( 36.8)	49( 21.0)	
undetermined	5( 1.2)	4( 1.7)	1( 0.5)	
DFS in month				
< 12	69( 16.5)	35( 18.9)	34( 14.6)	*0.403*
< 24	115( 27.5)	50( 27.0)	65( 27.9)	
< 36	83( 19.9)	39( 21.1)	44( 18.9)	
< 48	59( 14.1)	20( 10.8)	39( 16.7)	
≥ 48	92( 22.0)	41( 22.2)	51( 21.9)	

*BC* Breast cancer, *DM* Distant metastasis, *std* standard deviation, *IDC* Invasive ductal carcinoma, *LVI* Lymphovascular invasion, *HR* Hormone receptor, *HER2* Human epidermal growth factor receptor 2, *RT* Radiotherapy, *ET* Endocrine therapy, *DFS* Disease-free survival

^a^*P* value from ANOVA or Chi-square test on patients without the ‘unknown’ or ‘undetermined’ value

^b^ FISH test was mandatory for HER2 IHC 2 + tumor after 2010

### Comparison of patient characteristics between two subgroups

**Fig. 1:**
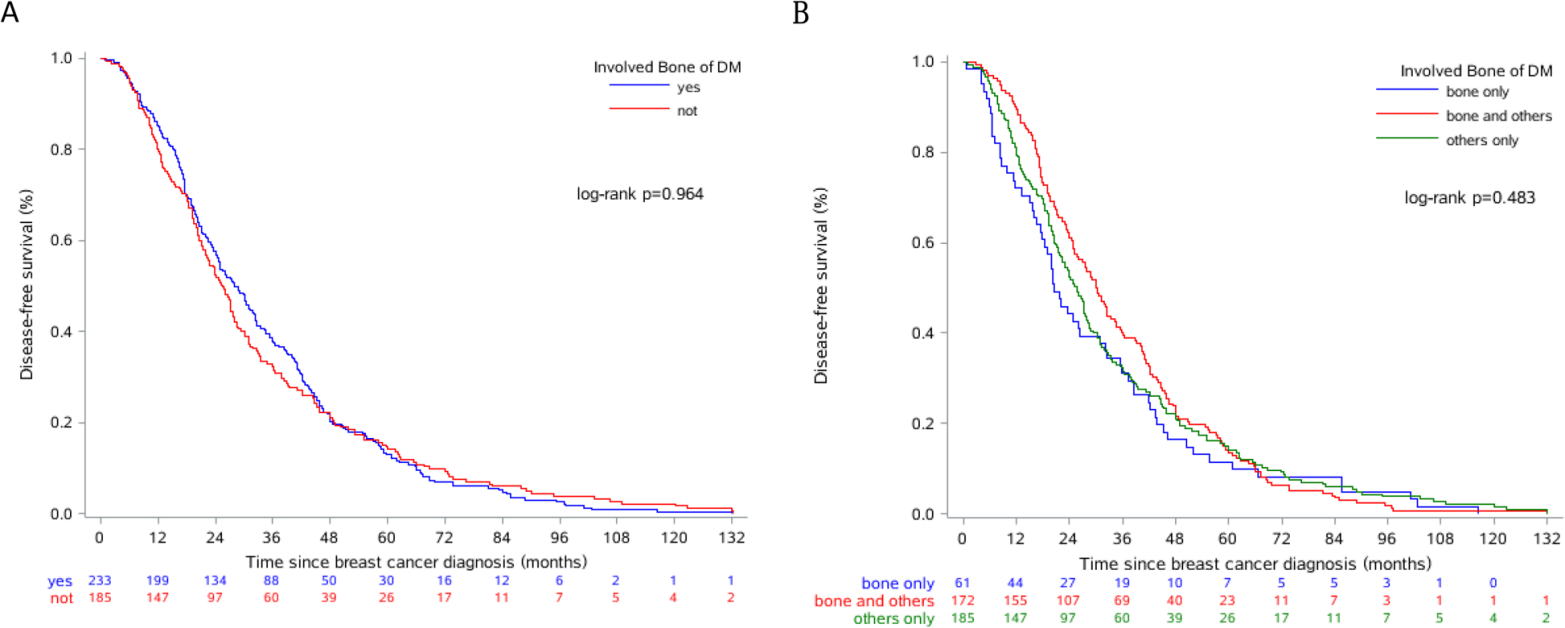
The Kaplan–Meier disease-free survival curves for patients grouped by bone involvement status at distant metastasis (DM)

Table 1 also indicates that most patient characteristics were not statistically different between the two subgroups (no and yes) within bone involvement (*p* =*0.181 – 0.992*) except for the variables HR (*p* =*0.001*) and HR-based ET compliance status (*p* =*0.004*). The patients with metastatic bone had significantly higher percentages of HR+ and full ET compliance compared to patients without. Such independent relationships should be verified in multivariate analysis. However, HR status of tumor should be regarded as the background and bone metastasis as the disease progression. Additionally, both categorical (*p* = *0.403*) and continuous DFS variables were compared between the two subgroups of bone involvement. Figure 1 shows that no statistical difference of their DFS medians were found between either two or among three patient subgroups of bone involvement status via the Kaplan-Meier curves (log-rank test *p* = *0.963, 0.483*).

### Logistic regression analysis

Table 2 shows the result of both univariate and multivariate analysis. It indicates that compared with HR− patients, the HR+ patients were independently and significantly associated with more than two times the higher risk of bone involvement (adjusted OR [aOR] = 2.317 – 2.329, *p* =*0.003, 0.004* , HR+ patients with non/partial or full ET compliance), regardless of ET compliance status. Such relationship strength was not influenced by the ET compliance degree (the full *vs.* non or partial, aOR = 1.006, *p* =*0.926*). In addition, the HER2+ patients were found to have no difference in risk of bone involvement (aOR = 0.827, *p* =*0.431*) from the HER2− patients. No other variables such as age, T and N stage, tumor grade, lymphovascular invasion (LVI), chemotherapy, or RT were found to have a significant OR (all *p* > *0.115*) either.

**Table 2:** Logistic regression on bone involvement

Variable	Class	Univariate			Multivariate ^a^		
		OR	95%CI	*P*	OR	95%CI	*P*
BC age (years)	< 40	1.575	( 0.786- 3.157)	*0.201*	1.280	( 0.589- 2.741)	*0.525*
	< 50	1.415	( 0.746- 2.686)	*0.288*	1.118	( 0.543- 2.300)	*0.762*
	< 60	1.189	( 0.624- 2.265)	*0.599*	1.065	( 0.521- 2.175)	*0.863*
	≥ 60	1.000		*ref*	1.000		*ref*
T stage ^b^	T1	1.000		*ref*	1.000		*ref*
	T2	0.883	( 0.546- 1.429)	*0.614*	0.827	( 0.492- 1.392)	*0.475*
	T3-4	0.943	( 0.515- 1.727)	*0.850*	0.833	( 0.423- 1.638)	*0.596*
N stage ^b^	N0	1.000		*ref*	1.000		*ref*
	N1-3	0.805	( 0.531- 1.219)	*0.306*	0.892	( 0.539- 1.476)	*0.656*
Pathology	IDC	1.120	( 0.675- 1.895)	*0.661*	1.149	( 0.491- 2.688)	*0.749*
	other	1.000		*ref*	1.000		*ref*
Tumor grade	I—II	1.000		*ref*	1.000		*ref*
	III	1.113	( 0.685- 1.809)	*0.666*	1.013	( 0.595- 1.726)	*0.962*
	unknown	1.845	( 0.486- 1.469)	*0.550*	0.598	( 0.232- 1.537)	*0.285*
LVI	yes	0.965	( 0.551- 1.690)	*0.902*	1.106	( 0.605- 2.019)	*0.744*
	no	1.000		*ref*	1.000		*ref*
	unknown	0.981	( 0.614- 1.567)	*0.935*	1.115	( 0.641- 1.940)	*0.699*
Chemotherapy	yes	1.273	( 0.495- 3.274)	*0.617*	2.015	( 0.603- 6.740)	*0.255*
	no	1.000		*ref*	1.000		*ref*
RT	yes	1.115	( 0.756- 1.644)	*0.584*	1.096	( 0.690- 1.741)	*0.699*
	no	1.000		*ref*	1.000		*ref*
HR/ET compliance	HR + /full	2.203	( 1.358- 3.574)	*0.001*	2.329	( 1.316- 4.124)	*0.004*
	HR + /non, partial	2.095	(1.264- 3.472)	*0.004*	2.317	(1.330- 4.036)	*0.003*
	HR −	1.000		*ref*	1.000		*ref*
HER2	positive	0.787	( 0.518- 1.198)	*0.264*	0.827	( 0.516- 1.327)	*0.431*
	negative	1.000		*ref*	1.000		*ref*
	undetermined	0.523	( 0.254- 1.077)	*0.079*	0.534	( 0.245- 1.166)	*0.115*

*DM* Distant metastasis, *BC* Breast cancer, *ref.* reference, *OR* Odd ratio, *CI* Confidence interval, *DFS* Disease-free survival, *IDC* Invasive ductal carcinoma, *LVI* Lymphovascular invasion, *RT* Radiotherapy, *HR* Hormone receptor, *HER2* Human epidermal growth factor receptor 2, *ET* Endocrine therapy

^a^ Final covariates included BC diagnosis age, diagnosis calendar year after 2010, tumor laterality, tumor quadrant, T stage, N stage, tumor pathology, tumor grade, LVI, chemotherapy, RT, and categorical disease-free time

^b^ Patients who had missing or unknown value were included in models, however, their ORs with insignificant *p* values were not tabulated

### Stratified analysis by HR or HER2 status

**Fig. 2:**
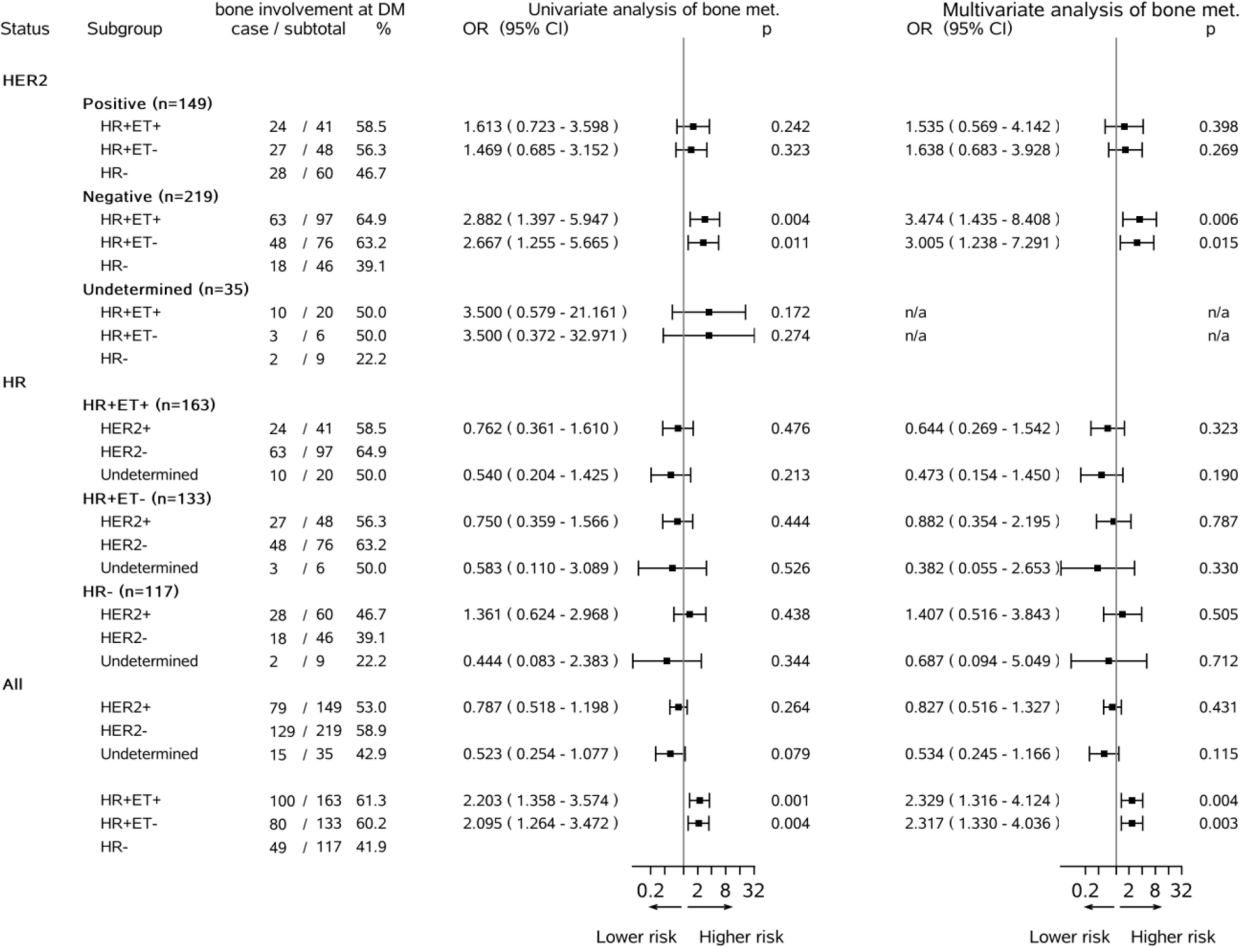
Stratified logistic regression analysis on bone involvement at distant metastasis (DM). *HR* + */ − *hormone receptor-positive/negative, *HER2* + */ − *human epidermal growth factor receptor 2-positive/negative, *ET* + */ − *endocrine therapy full/non or partial compliance status. The final covariates included BC diagnosis age, diagnosis calendar year after 2010, tumor laterality, tumor quadrant, T stage, N stage, tumor pathology, tumor grade, LVI, chemotherapy, RT, and categorical disease-free time

To further understand the association of HR or HER2 status with bone involvement, both stratified univariate and multivariate logistic regressions were conducted. Figure 2 illustrates the results of these stratified analyses and ones from the overall population analysis. These results further indicated that the significant associations of HR+ with bone involvement were found to be strongly and significantly existing among the HER2− patients (*n* = 219, aOR = 3.474, 3.005; *p* = *0.006, 0.015*) but not among the HER2+ patients (*n* =149, aOR =1.535, 1.638; *p* = *0.398, 0.269*). HR+ patients still had more than thrice the risk of bone involvement compared with HR− patients among the HER2− patients. The smaller sample size of the HER2+ patients might have influenced the result significance of the results. Again, the similar ORs of bone involvement related to the ET compliance status at all stratified analysis (all *p* > *0.05*) were found to pinpoint the uninfluential role of the ET compliance. No significant relationship of HER2+ *vs.* HER2− with bone involvement were found at the three patient subgroups defined by the HR and ET compliance statuses (*n* = 163, 133, 117).

## Discussion

BM is the most common DM of BC [1, 2]. It is challenging to determine the independent risk relationships of HR and HER2 status per se with BM given the involvement of ET and HER2 targeted agents. In this retrospective study, we analyzed 481 Chinese BC patients with first DM over 11 years. All of them did not have any HER2 targeted therapy due to the lag approval and high cost in China compared with the developed countries. After the adjustment of ET compliance status in HR+ patients, the study’s results showed that HR+ was significantly associated with higher risk of bone involvement, while HER2 status per se was not. The different ET compliance status did not impact the development of BM in HR+ patients. Through the multivariate analysis, many factors like age, T stage, N stage, pathology, tumor grade, LVI, chemotherapy, and RT were found not related with BM. In our view, although this finding is not a particularly novel one, this study population has been generally less studied in the literature. The study results would help the new research hypothesis generation and better guide the imaging use for BM detection in patient subgroups.

BM is a complex biologic interactive process of tumor seeds and soil factors at bone [2, 8, 9]. The relationship of ER+ or HR+ with higher risk of BM has been established in many studies [10–12, 15]. Liede et al. examined 257 (13.2%) BM patients among 2,097 stage I - III BC patients in their follow-up period (median = 12.5 years) to show that bone as the first metastasis site was associated with ER+ (Hazard Ratio [HR] = 1.85, *p* =*0.008*), but not with PR+ (HR = 0.79, *p* =*0.20*) through multivariate analysis [10]. Similarly, Bartmann et al. analyzed 886 BC patients with first DM among 9,625 BC patients with a median follow-up of 53 months. He found that the highest BM rate was among HR+/HER2− patients (66.8 ~ 70.1%) followed by HR+/HER2+ (64.1%), HR−/HER2+ (45.6%), and HR−/HER2− (38.9%) patients (*p* <*0.001*) [11]. Parkes et al. analyzed 1,048 BC patients with bone-only metastasis as the first site of metastasis at MD Anderson Cancer Center and also found that HR+/HER2− subtype contributed to the majority (78%), followed by 11% in HR+/HER2+, 7% in HR−/HER2− and 3% in HR−/HER2+ [12]. Paluch-Shimon et al. studied 137 metastatic HER2+ BC patients and similarly showed that compared to HR− (*n* = *81*) patients, HR+ patients (*n* = *56*) had a trend for more bone metastasis (49% vs. 34%, *p* = *0.10*) [14]. In general, our results are in agreement with most published literature.

HR+ has long been regarded as a risk factor for BM. However, how the ET impacts this link has rarely been studied [10–12]. Liede et al. found that Tamoxifen was associated with a trend of lower risk of first BM (HR = 0.74, 95%CI 0.53 – 1.03, *p* = *0.07*) through multivariate analysis [10]. The non/partial compliance of ET in HR+ patients has been known to be associated with higher recurrence and mortality of BC patients [16]. In our study, we examined the relationship of the ET compliance status with first BM risk in HR+ patients. The study result indicated that the ET compliance had an insignificant impact on the development of BM in HR+ patients.

The relationship of HER2+ and BM is still inconclusive [10, 17, 18]. In this study, all HER2+ patients did not receive the HER2 targeted therapy. Therefore, the therapeutic effect on the relationship between HER2 status and BM was not investigated. In fact, most studies investigated the BM risk difference among the four patient subtypes formed by HR and HER2 status and did not elaborate on the confounding effect of the HER2 targeted therapy in analysis [10, 17]. Park et al. analyzed 313 Korean women with BC in 1994 – 2000 with a median follow-up of 93 months and found that BM was most commonly in HR+/HER2− (54.3%), HR+/HER2+ (50.0%), HR−/HER2+ (42.9%) and least in HR−/HER2− (12.5%) patients (*p* = *0.04*) [17]. In their study, the use of HER2 targeted agents was not mentioned nor investigated in the study [17]. However, Liede et al. found that HER2+ (vs. HER2−) was not associated with first BM (HR = 1.25, 95%CI 0.90 – 1.74, *p* = *0.18*) through multivariate analysis [10]. Hess et al. studied 11,011 stage I – III BC patients, and found that there were different 12-year cumulative metastasis rates - 23% for bone, 15% for liver, 14% for lung, 13% for distant lymph, 10% for brain, and 8% for pleura. In their study, the effect of HER2+ on BM did not depend on HR status, but changed significantly over time (competing HR = 1.4, 95%CI 1.1 - 1.7 in HR+ patients, HR= 0.7 95%CI 0.6 – 1.0 in HR− patients at the period of 0 - 4 years since diagnosis) [18]. In their study, the competing HR of HER2+ (*p* value) was 0.49 (*p* < *0.0001*) for BM [18]. In our opinion, the increasing therapeutic effect of HER2 targeted therapy on tumor control over time could be significantly impacting the relationships of HER2+ per se on BM. However, Parkes et al. analyzed the HER2+ BC patients (*n* = 30) and found that bone, visceral, soft tissue and CNS metastasis did not significantly differ between who had and who did not have Trastuzumab (p = *0.144*) [19]. Indeed, any variation in therapeutics regimens, stage of primary tumor, geographical regions, time periods, and subtype criteria could contribute to the inconsistency of BM relationships with the HR and HER2 status across different studies.

This retrospective study has strengths. First, the logistic regression was intentionally chosen to estimate the cross-sectional relationship in order to avoid the influence of BM diagnosis which was sensitive to the imaging timing. In this regard, a categorical DFS was created and treated as a covariate at the multivariate model. Second, the analyzed patients did not have any HER2 targeted therapy, nor take any OFS procedure or drugs, nor use any bone modifying agents. Third, two ET compliance statuses were analyzed to adjust for possible different effects of ET dosing on BM in HR+ patients. Lastly, the stratified analyses have further demonstrated that the subset HER2− patients had exemplified the significant relationship of HR+ and bone involvement.

Like other retrospective studies, this study has limitations. This included the data recall bias especially on ET, patients from a single cancer center, lack of one standard imaging tool for DM diagnosis, and lack of adequate and high quality data of BM features like bone site and metastasis type (e.g. osteolytic *vs.* osteogenic *vs*. mixed one) for additional analysis. More relevantly, the study population lacked the anti-HER2 therapy, which now is a routine for HER2+ patients. The extrapolation of the study finding may not be applicable in the presence of anti-HER2 therapy. However, the study results were from the real-world data and should be considered having applicable values to the clinical practice for some patients.

### Clinical implications

The study finding would assist the hypothesis generation of clinical research and provide some guidance of better using bone imaging and bone modifying agents in BC patients. Both HR and HER2 status should be considered along with bone symptoms, labs and physical exam for the BM surveillance and possible prevention through drug intervention.

## Conclusions

HR+ tumors are significantly associated with bone metastasis mainly in HER2− BC patients. ET appears to have little impact on this link. HER2 status per se is not associated with risk of first BM.

## Data Availability

The datasets generated during and analyzed during the current study are not publicly available due to the confidentiality agreement and research data policy of the Fourth Hospital of Hebei Medical University, but are available from the corresponding author on reasonable request.

## Abbreviations

ANOVA: Analysis of variance
aOR: Adjusted odd ratio
BC: Breast cancer
BM: Bone metastasis
CI: Confidence interval
DFS: Disease-free survival
DM: Distant metastasis
ER: Estrogen receptor
ER +: Estrogen receptor-positive
ET: Endocrine therapy
FISH: Fluorescence in situ hybridization
HER2: Human epidermal growth factor receptor 2
HER2 −: Human epidermal growth factor receptor 2-negative
HER2 +: Human epidermal growth factor receptor 2-positive
HR: Hazard Ratio
HR: Hormone receptor
HR −: Hormone receptor-negative
HR +: Hormone receptor-positive
IDC: Invasive ductal carcinoma
LRR: Locoregional recurrence
LVI: Lymphovascular invasion
OFS: Ovarian function suppression
OR: Odd ratio
PR: Progesterone receptor
PR +: Progesterone receptor-positive
RT: Radiotherapy
std: Standard deviation

## References

[1] Kennecke H, Yerushalmi R, Woods R (2010). Metastatic behavior of breast cancer subtypes. J Clin Oncol.

[2] Coleman RE, Croucher PI, Padhani AR (2020). Bone metastases. Nat Rev Dis Primers.

[3] Ahn SG, Lee HM, Cho SH (2013). Prognostic factors for patients with bone-only metastasis in breast cancer. Yonsei Med J.

[4] Burstein HJ, Lacchetti C, Anderson H (2019). Adjuvant endocrine therapy for women with hormone receptor-positive breast cancer: ASCO clinical practice guideline focused update. J Clin Oncol.

[5] Ce C (2015). Adjuvant bisphosphonate treatment in early breast cancer: meta-analyses of individual patient data from randomised trials [published correction appears in Lancet. 2016 Jan 2;387(10013):30] [published correction appears in Lancet. 2017 Jun 24;389(10088):2472]. Lancet.

[6] Coleman R, Cameron D, Dodwell D (2014). Adjuvant zoledronic acid in patients with early breast cancer: final efficacy analysis of the AZURE (BIG 01/04) randomised open-label phase 3 trial. Lancet Oncol.

[7] Early Breast Cancer Trialists’ Collaborative group (EBCTCG) (2021). Trastuzumab for early-stage, HER2-positive breast cancer: a meta-analysis of 13 864 women in seven randomised trials. Lancet Oncol.

[8] Pulido C, Vendrell I, Ferreira AR (2017). Bone metastasis risk factors in breast cancer. Ecancermedicalscience.

[9] Salvador F, Llorente A, Gomis RR (2019). From latency to overt bone metastasis in breast cancer: potential for treatment and prevention. J Pathol.

[10] Liede A, Jerzak KJ, Hernandez RK, Wade SW, Sun P, Narod SA (2016). The incidence of bone metastasis after early-stage breast cancer in Canada. Breast Cancer Res Treat.

[11] Bartmann C, Wischnewsky M, Stüber T (2017). Pattern of metastatic spread and subcategories of breast cancer. Arch Gynecol Obstet.

[12] Parkes A, Clifton K, Al-Awadhi A (2018). Characterization of bone only metastasis patients with respect to tumor subtypes. NPJ Breast Cancer.

[13] Fan L, Strasser-Weippl K, Li JJ (2014). Breast cancer in China. Lancet Oncol.

[14] Liao N (2016). HER2-positive breast cancer, how far away from the cure?-on the current situation of anti-HER2 therapy in breast cancer treatment and survival of patients. Chin Clin Oncol.

[15] Paluch-Shimon S, Ben-Baruch N, Wolf I (2009). Hormone receptor expression is associated with a unique pattern of metastatic spread and increased survival among HER2-overexpressing breast cancer patients. Am J Clin Oncol.

[16] Visvanathan K, Fabian CJ, Bantug E (2019). Use of endocrine therapy for breast cancer risk reduction: ASCO clinical practice guideline update. J Clin Oncol.

[17] Park HS, Kim S, Kim K (2012). Pattern of distant recurrence according to the molecular subtypes in Korean women with breast cancer. World J Surg Oncol.

[18] Hess KR, Esteva FJ (2013). Effect of HER2 status on distant recurrence in early stage breast cancer. Breast Cancer Res Treat.

[19] Parkes A, Warneke CL, Clifton K (2018). Prognostic factors in patients with metastatic breast cancer with bone-only metastases. Oncologist.

